# Platelet Count of Zero: A Case of Vancomycin-Induced Immune Thrombocytopenia

**DOI:** 10.7759/cureus.94454

**Published:** 2025-10-13

**Authors:** Shiv B Gakhar, Adam Hurwitz, Colton Sellars, Andrew Willinger, Melanie Cheing

**Affiliations:** 1 Internal Medicine, HCA Florida Largo Hospital, Largo, USA; 2 Pulmonary and Critical Care, HCA Florida Largo Hospital, Largo, USA

**Keywords:** drug-induced thrombocytopenia, immune thrombocytopenia purpura, plasmapheresis, platelets, vancomycin therapy

## Abstract

Immune thrombocytopenia (ITP) is an acquired hematologic disorder marked by immune-mediated destruction of platelets, leading to profound thrombocytopenia and increased bleeding risk. Drug-induced immune thrombocytopenia (DITP), specifically vancomycin-induced immune thrombocytopenia (VIIT), is a rare but serious adverse drug reaction, caused by drug-dependent antibodies that accelerate platelet clearance.

We present a case of a 72-year-old male who developed life-threatening thrombocytopenia, with a nadir of <1 K/µL, following re-exposure to vancomycin. The decline began within three days of treatment reinitiation and persisted despite platelet transfusions, corticosteroids, and intravenous immunoglobulin (IVIG). A diagnosis of VIIT was made after excluding other etiologies, including heparin-induced thrombocytopenia (HIT), thrombotic microangiopathy, and sepsis-associated thrombocytopenia. Platelet counts remained refractory until plasmapheresis and drug discontinuation, with recovery to 71 K/µL within one week. This case highlights the importance of early recognition of VIIT and suggests a potential role for plasmapheresis in refractory cases. Prompt drug discontinuation is critical for recovery.

## Introduction

Immune thrombocytopenia (ITP) is an acquired hematologic disorder characterized by isolated thrombocytopenia due to antibody-mediated platelet destruction. While many cases are idiopathic, secondary causes, such as medications, must be considered. Vancomycin-induced immune thrombocytopenia (VIIT) is a rare but potentially life-threatening drug reaction. It is caused by vancomycin-dependent, platelet-reactive antibodies that accelerate platelet clearance [1]. The estimated incidence is 3.3% among patients treated with intravenous vancomycin, although it is likely underrecognized due to its similarity to other thrombocytopenic syndromes [2]. The rapidity and severity of thrombocytopenia in VIIT can pose significant diagnostic and therapeutic challenges. Treatment usually consists of vancomycin discontinuation, corticosteroids, and intravenous immunoglobulin (IVIG), with plasmapheresis reserved for refractory cases to help clear circulating immune complexes.

We present a case of vancomycin-associated ITP in a 72-year-old male with recent surgery who developed life-threatening thrombocytopenia, with platelet counts dropping to <1 K/µL after re-exposure to vancomycin.

## Case presentation

**Figure 1:**
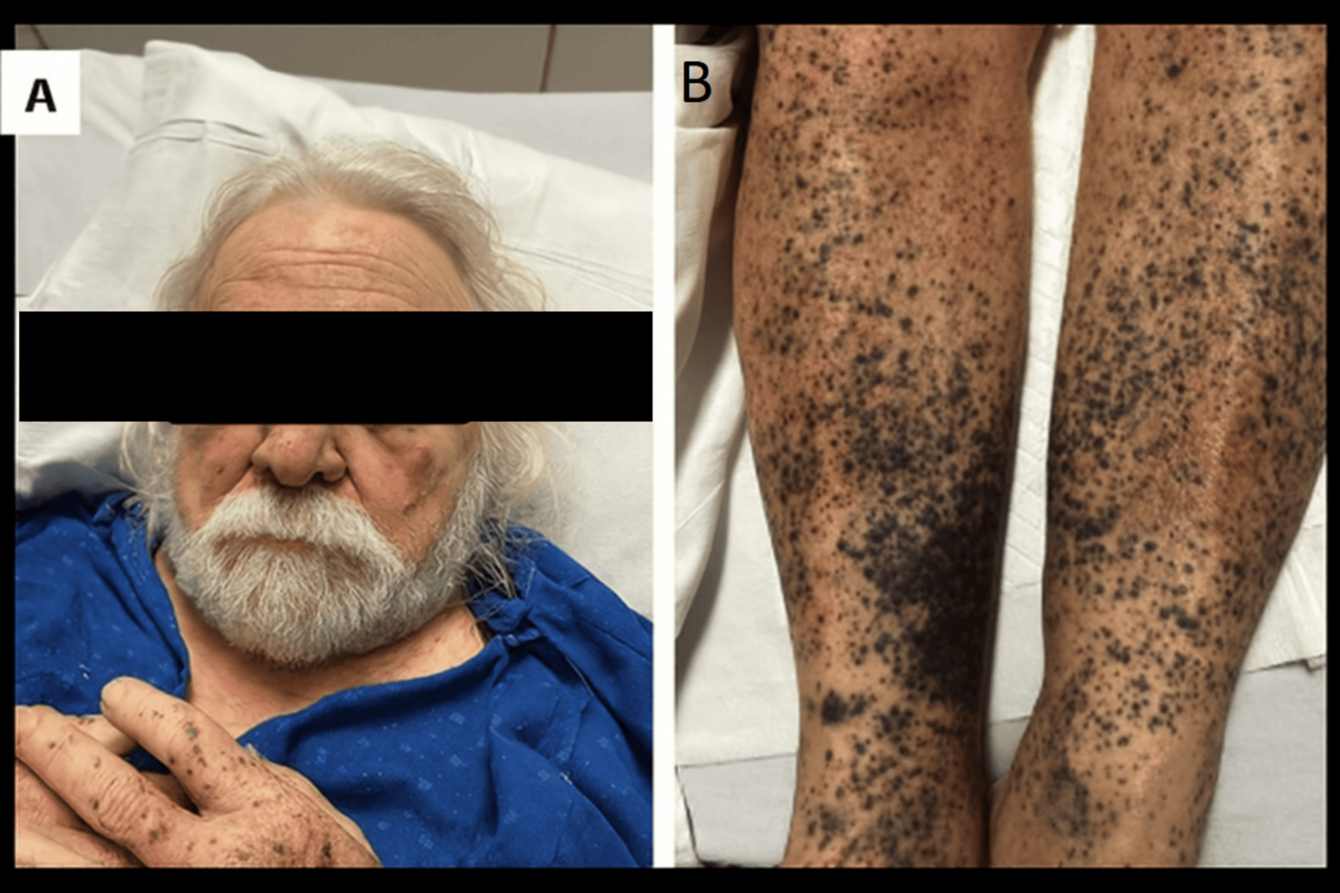
Clinical Purpuric and Petechial Findings (A) Face: Mild bilateral periorbital ecchymosis, with small areas of non-blanching purple spots. Eyes and eyebrows have been obscured to maintain patient confidentiality. (B) Bilateral lower extremities: Extensive, diffuse purpuric and petechial rash on the lower legs, more pronounced distally around the ankles and lower shins, with some areas of confluence forming large ecchymotic patches. The lesions are non-blanching, vary in size, and exhibit dark purple discoloration. The patient provided written and signed consent, allowing publication of this identifiable facial image in an open-access journal.

A 72-year-old male with hypertension, hepatitis C (treated), liver transplant in 2012 (off immunosuppressants), and recent colorectal surgery, presented from a skilled nursing facility with urinary retention and abdominal discomfort. One month earlier, he had undergone open low anterior resection for a rectosigmoid tubulovillous adenoma. His postoperative course was complicated by urinary retention, requiring Foley catheterization and discharge with the catheter in place (Figure 1).

On hospital presentation, computed tomography (CT) of the abdomen and pelvis revealed postsurgical changes and fat stranding consistent with cellulitis. CT angiography showed pulmonary emboli in the right lower lobe, with mild right ventricular dilation. He was admitted to progressive care and started on intravenous vancomycin and cefepime for a presumed surgical site infection. Anticoagulation was initiated with heparin but was discontinued due to dropping platelets, then switched to apixaban.

The patient initially received one dose of IV vancomycin, followed by three days of oral vancomycin, then ceftriaxone and metronidazole. On readmission, IV vancomycin was restarted for four days. The final dose coincided with a platelet count drop from 328 to 217 K/µL. His first vancomycin exposure occurred 21 days prior to thrombocytopenia onset, suggesting sensitization.

Within 96 hours after reinitiation, platelets fell rapidly from 328 to 217, then to 3 and 0 K/µL. Anticoagulation was stopped, and he received seven leukoreduced platelet transfusions with minimal response. The exam showed diffuse petechiae, purpura on the extremities and trunk, and bilateral periorbital ecchymosis without mucosal bleeding. Labs revealed normal lactate dehydrogenase (LDH), bilirubin, haptoglobin, aspartate aminotransferase/alanine aminotransferase (AST/ALT), and A disintegrin and metalloproteinase with thrombospondin motifs 13 (ADAMTS13). Peripheral smear showed no schistocytes or thrombotic microangiopathy. Disseminated intravascular coagulation was excluded. Heparin-induced thrombocytopenia (HIT) testing was negative, and the timing made HIT unlikely (Table 1).

**Table 1:** Lab Results Laboratory results obtained during hospitalization are presented with corresponding reference ranges. “Normal” indicates values within the stated reference range. Platelet counts are shown in K/µL (×10³/µL). LDH, lactate dehydrogenase; AST, aspartate aminotransferase; ALT, alanine aminotransferase; ADAMTS13, A disintegrin and metalloproteinase with thrombospondin motifs 13; HIT, heparin-induced thrombocytopenia

Parameter	Patient Value	Reference Range
Platelet count (K/µL) - baseline	328	150-450
Platelet count - day 2	217	150-450
Platelet count - nadir	<1	150-450
LDH (U/L)	Normal	140-280
Total bilirubin (mg/dL)	Normal	0.1-1.2
Haptoglobin (mg/dL)	Normal	30-200
AST (U/L)	Normal	10-40
ALT (U/L)	Normal	7-56
ADAMTS13 activity (%)	Normal	>67
Peripheral smear	No schistocytes	-
HIT antibody	Negative	Negative

Despite high-dose corticosteroids and four doses of IVIG (1 g/kg), platelets remained critically low. He was transferred to the Intensive Care Unit and underwent three sessions of plasmapheresis. Platelet count gradually rose to 71 K/µL within seven days of vancomycin discontinuation. He was transitioned to a tapering oral prednisone dose and discharged in stable condition, with hematology follow-up. No further bleeding or platelet drops occurred.

Written informed consent for publication of clinical details and identifying images, including Figure 1A, was obtained from the patient, and a signed consent statement has been provided to the journal.

## Discussion

DITP is a rare but serious complication of over 300 medications, including vancomycin [2]. DITP typically presents with rapid thrombocytopenia and must be differentiated from HIT, TTP, sepsis-associated thrombocytopenia, and bone marrow suppression. In this case, HIT testing was negative, ADAMTS13 activity was normal, and the peripheral smear showed no schistocytes. Sepsis-related thrombocytopenia was unlikely, given the patient’s afebrile status, stable hemodynamics, and absence of leukocytosis at the time of nadir.

Vancomycin follows a “quinine-type” mechanism, where drug-dependent antibodies bind platelet surface glycoproteins (GPIb/IX and GPIIb/IIIa). This binding requires vancomycin presence, forming immune complexes cleared by the reticuloendothelial system, causing abrupt platelet destruction [3]. Diagnosis of VIIT is clinical, supported by drug timing, rapid platelet drop, and recovery after cessation. In this case, the patient developed profound thrombocytopenia (<1 K/µL) within three days of vancomycin re-exposure, consistent with immune memory from prior exposure, three weeks earlier.

Platelet transfusions are often ineffective because transfused platelets are rapidly destroyed by circulating antibodies. Corticosteroids and IVIG likewise often fail to produce significant improvement. A study by Von Drygalski et al. showed that 11 of 14 patients receiving platelet transfusions had no response, illustrating therapy limitations in VIIT [4].

In this report, the patient responded only after plasmapheresis. Although vancomycin was stopped, improvement likely reflects the removal of circulating immune complexes or delayed drug clearance. The patient’s urinary retention may have prolonged vancomycin exposure, further contributing to the severity [5].

This case highlights the need to recognize VIIT early. Prompt vancomycin discontinuation is essential. In refractory cases with a nadir <1 K/µL or active bleeding, plasmapheresis may be a valuable adjunct. Given vancomycin’s widespread use, awareness of this rare but serious complication is crucial to reduce morbidity and mortality.

## Conclusions

We present a case of severe VIIT that was refractory to corticosteroids, IVIG, and platelet transfusions, ultimately requiring plasmapheresis for recovery. This case highlights the rapid onset and profound severity of thrombocytopenia following re-exposure to vancomycin. Our discussion emphasizes the importance of recognizing VIIT early and differentiating it from other causes, such as HIT, TTP, or sepsis-related thrombocytopenia. Similar to other DITPs, VIIT may present with minimal response to conventional therapies, underscoring the need for timely discontinuation of the offending agent. The successful use of plasmapheresis in our patient suggests a potential therapeutic role in refractory cases. Clinicians should maintain high suspicion for VIIT in patients with precipitous platelet declines after re-exposure to vancomycin.
